# IFI16 Induction by Glucose Restriction in Human Fibroblasts Contributes to Autophagy through Activation of the ATM/AMPK/p53 Pathway

**DOI:** 10.1371/journal.pone.0019532

**Published:** 2011-05-05

**Authors:** Xin Duan, Larissa Ponomareva, Sudhakar Veeranki, Divaker Choubey

**Affiliations:** 1 Department of Environmental Health, University of Cincinnati, Cincinnati, Ohio, United States of America; 2 Cincinnati VA Medical Center, Cincinnati, Ohio, United States of America; Roswell Park Cancer Institute, United States of America

## Abstract

**Background:**

Glucose restriction in cells increases the AMP/ATP ratio (energetic stress), which activates the AMPK/p53 pathway. Depending upon the energetic stress levels, cells undergo either autophagy or cell death. Given that the activated p53 induces the expression of IFI16 protein, we investigated the potential role of the IFI16 protein in glucose restriction-induced responses.

**Methodology/Principal Findings:**

We found that glucose restriction or treatment of human diploid fibroblasts (HDFs) with the activators of the AMPK/p53 pathway induced the expression of IFI16 protein. The induced levels of IFI16 protein were associated with the induction of autophagy and reduced cell survival. Moreover, the increase in the IFI16 protein levels was dependent upon the expression of the functional ATM protein kinase. Importantly, the knockdown of the IFI16 expression in HDFs inhibited the activation of the ATM/AMPK/p53 pathway in response to glucose restriction and also increased the survival of HDFs.

**Conclusions/Significance:**

Our observations demonstrate a role for the IFI16 protein in the energetic stress-induced regulation of autophagy and cell survival. Additionally, our findings also indicate that the loss of IFI16 expression, as found in certain cancers, may provide a survival advantage to cancer cells in microenvironments with low glucose levels.

## Introduction

Ataxia Telangiectasia (A-T) is an inherited disorder [1], [2]. The clinical presentations of the AT are due to an autosomal recessive mutation in the Ataxia Telangiectasia (*ATM*) gene. The ATM protein is a ∼370 kDa Ser/Thr kinase, which is localized in the cytoplasm and nucleus [2]. In response to oxidative stress, ATM protein is phosphorylated at Ser-1981 [1], [2]. This phosphorylation activates the kinase activity, which results in phosphorylation of its substrates, including the AMP-activated protein kinase-α (AMPKα) and p53 [2], [3]. Interestingly, activation of the ATM kinase in the cytoplasm regulates autophagy through activation of AMPK [4], [5].

AMPKα, a highly conserved and widely expressed protein kinase, acts as an intracellular energy sensor [6], [7]. Reduced levels of glucose in normal eukaryotic cells, which increase the AMP levels (increases the AMP/ATP ratio), activate the AMPKα through phosphorylation of Thr-172 and also increase its levels [7]. Several protein kinases can phosphorylate the AMPKα including the LKB1 [8], ATM [3], and calmodulin-dependent protein kinase kinase-β [9]. However, in response to energetic stress, AMPKα is phosphorylated in an ATM-dependent and LKB1-independent manner [3]. Studies have suggested that the activation of AMPKα inhibits cell proliferation in both tumor and nonmalignant cells [10]. Furthermore, a persistent activation of AMPKα potentiates the p53-dependent cellular senescence in mouse embryonic fibroblasts (MEFs) [11].

p53 tumor suppressor is activated in cells in response to a variety of stimuli, including DNA damage, hypoxia, oxidative stress, and the energetic stress [12], [13]. The activation of AMPK in response to energetic stress induces phosphorylation of p53 at Ser-15 residue [11], [14], resulting in its activation and stabilization, which increases the p53 protein levels [12]. The activated p53 binds to its DNA-binding consensus sequence that is present in its target genes [15]. The binding of p53 to its target genes activates the transcription of genes, such as *p21* [16] and *IFI16* [17], and repress the transcription of certain anti-apoptotic genes [15]. The p53 regulate glucose cellular metabolism [18] and autophagy [19].

It has been shown that MEFs treated with low glucose concentrations arrest in the G_1_ phase of the cell cycle [11]. The arrest depends on the activation of AMPK and phosphorylation of p53 at Ser-15 [11]. Accordingly, the p53-deficient cells fail to arrest under low glucose conditions [11]. A study has noted that glucose deprivation of mouse thymocytes and human osteosarcoma cell line U2OS induces phosphorylation of AMPKα and promotes a p53-dependent decrease in cell survival [14]. Notably, glucose deprivation of U2OS cells increases the p53 mRNA and protein levels, and the phosphorylation at Ser-46 [14].

Autophagy involves the degradation of cellular components [20]. The autophagy is regulated by proteins (encoded by autophagy-related genes or the *ATG* genes), which participate in the formation of autophagosomes. Under glucose restriction, induction of autophagy is thought to provide a nutrient source and promote cell survival [20], [21]. However, under severe glucose restriction, which results in higher energetic stress, autophagy may lead to cell death [22]. Consistent with this idea studies indicate that autophagy may operate upstream of apoptosis [21], [22]. Importantly, autophagy regulates infection [23]. Additionally, autophagy modulates inflammation by activating an inflammasome activity and by targeting the pro-IL-1β for degradation [24], [25].

The interferons (IFN) and p53 activate the transcription of the *IFI16* gene (encoding for the IFI16 protein), a member of the *IFI200-*genes family [26], [27]. The gene encodes for three isoforms (A, B, and C) of the IFI16 protein through an alternative splicing of mRNA and the B isoform of the protein is predominant in HDFs [27]. Upon sensing cytosolic double-stranded DNA (dsDNA), the IFI16 protein induces the expression of the IFN-β [28]. The extent of the cytoplasmic and nuclear localization of the IFI16 protein depends on the cell type [27]. Increased levels of the IFI16 protein in human primary cells, including the human diploid fibroblasts (HDFs), are associated with the onset of cellular senescence [29], [30]. Furthermore, the knockdown of IFI16 expression in HDFs results in down-regulation of p21^CIP1^ protein levels and delays the onset of cellular senescence [30]. Consistent with a role for the IFI16 protein in cellular senescence-associated cell growth arrest, increased levels of IFI16 protein up-regulate the expression of p21^CIP1^ and inhibit the E2F-mediated transcription [30]. Notably, ectopic co-expression of the IFI16 protein with p53 in MCF-7 cells enhances the transcription of the known p53 target genes, such as *p21*, *Hdm2*, and *Bax* [31]. Moreover, the knockdown of IFI16 expression in MCF-7 cells decreases the phosphorylation of p53 at Ser-15, following ionizing radiation treatment, and decreases the p53-mediated apoptosis [31]. IFI16 protein binds to p53 and increased expression of the IFI16 protein in cells potentiates the p53-mediated transcriptional activation of the target genes [32], [33].

Given that expression of the *IFI16* gene is transcriptionally activated by p53 [17], increased levels of the IFI16 protein in HDFs are associated with the onset of cellular senescence [30], and the energetic stress-induced activation of the AMPK/p53 pathway induces cellular senescence in MEFs [11], we investigated the potential role of the IFI16 protein in glucose restriction-induced activation of energetic stress. Here we report that the IFI16 protein is required for the activation of the ATM/AMPK/p53 pathway and autophagy upon glucose restriction.

## Materials and Methods

### Cell Lines, Culture Conditions, and Treatments

Normal human fetal lung fibroblasts WI-38 (AG06814N) at population doubling (PD) 15 (passage 12) and AT skin fibroblasts (AG03057) at PD 6 (passage 4) were obtained from the National Institute of Aging Cell Culture Repository (Coriell Medical for Medical Research, Camden, NJ). Both WI-38 and AT cell cultures were maintained (5.5% CO_2_ and ∼21% O_2_) in DMEM culture medium with high glucose (4.5 g/L; glucose concentration equivalent to ∼25 mM), which was supplemented with 10% fetal bovine serum and antibiotics (Invitrogen, Carlsbad, CA). Cell cultures were regularly split 1∶ 4 on approaching confluence. Thus, each cell passage was equivalent to ∼2 cell PDs. Sub-confluent cultures of HDFs, when indicated, were treated with the indicated reduced concentrations (from 1 mM to 0.25 mM; normal average glucose concentration in the human serum is ∼5 mM) of glucose by incubating in glucose and pyruvate-free DMEM medium (cat # 11966-025; Invitrogen) supplemented with 10% fetal bovine serum. When indicated, cells were treated with either dimethyl sulphoxide (DMSO; vehicle) alone or 3-methyladenine (from Sigma; 5 mM concentration) dissolved in DMSO for the indicated time.

HDFs were collected by trypsinization from cell culture plates and the number of viable cells in the cultures were counted (in triplicates) after Trypan Blue staining using Countess Automated Cell Counter (Invitrogen) and cell counting kit as suggested by the supplier.

### Plasmids and Expression Vectors

The wild-type IFI16-luc-reporter plasmid, which contains the promoter region (1.677 kb; 1,467 bp upstream of the transcriptional start site) of the *IFI16-*gene, linked a reporter gene, whose transcription can be activated by the p53, has been described [17].

### Knockdown of the Expression

Young WI-38 HDFs were either infected with control shRNA particles (sc-108080; from Santa Cruz Biotech, Santa Cruz, CA) or IFI16 shRNA Lentiviral particles (sc-35633-V, Santa Cruz) in a six well plate as suggested by the supplier. A day after infections, cells were selected with puromycin (1 µg/ml) for three days. The selected cells were pooled and maintained in puromycin (0.5 µg/ml) for another week. For experiments, cells were cultured without puromycin for at least three days. The selected cells were analyzed for the knockdown of IFI16 expression by quantitative PCR as well as immunoblotting. We were able to reproducibly achieve ∼70–90% knockdown of the IFI16 expression.

### Immunoblotting and Antibodies

To prepare cell lysates, cells were collected from plates in PBS and lysed using RIPA buffer supplemented with protease inhibitors and phosphatase inhibitors as described previously [17]. The cell lysates were sonicated briefly before centrifugation at 10,000 rpm in a microfuge for 10 min. The supernatants were collected and equal amounts of proteins were processed for immunoblotting. Antibodies specific for IFI16 (sc-8023) and p53 (sc-126) were purchased from Santa Cruz Biotech (Santa Cruz, CA). Antibodies specific for β-actin (cat # 4967), p-ATM (ser1981) (cat # 2873), ATM, (cat # 4526), p-AMPK (Thr172) (cat #2531), AMPK (cat # 2532), p-p53 (ser15) (cat # 9284), p-p53 (ser46) (cat # 2521) and p-p53 (ser392) (cat # 9281) were purchased from Cell Signaling Technology (Danvers, MA). Horseradish peroxidase (HRP) conjugated secondary anti-mouse (NXA-931) and anti-rabbit (NA-934) antibodies were from Amersham Biosciences.

HDFs were fractionated into the nuclear and cytoplasmic fractions as described previously [34]. The detection of the histone H3 in the nuclear fraction and the IκBα protein in the cytoplasmic fractions served as the quality control for the cell fractionations.

### Reverse Transcriptase Reaction, Real-Time PCR, and qPCR Array

Total RNA was isolated from WI-38 or AT fibroblasts with Trizol reagent (Invitrogen, Carlsbad, CA, USA). cDNA synthesis was done using primers with SuperScript First-strand Synthesis System for RT-PCR (Invitrogen, Carlsbad, CA, USA). Quantitative real-time TaqMan PCR technology (Applied Biosystems, Foster City, CA, USA) was used. The PCR cycling program consisted of denaturing at 95°C for 10 min and 40 cycles at 95°C for 15 seconds, and annealing and elongation at 60°C for 1 min. The TaqMan assays for *IFI16* (assay Id #Hs00194216_m1), *p53* (assay Id #Hs00153349 _m1), *p21* (assay Id #Hs00355782_m1), and for the endogenous control β-actin (assay Id# Hs99999903_ml) were purchased from Applied Biosystems (Foster City, CA) and used as suggested by the supplier.

To compare expression of pro- and anti-apoptotic genes between control HDFs and HDFs after the IFI16 expression knockdown under reduced glucose levels (0.25 mM), we isolated total RNA from HDFs incubated at either 25 mM or 0.25 mM glucose for 24 h. cDNA was synthesized as described above and equal amounts of cDNA was used to performed RT^2^ Profiler PCR Array using human apoptosis PCR array (PAHS-012A-2; from SA Bioscience, Fredrick, MD) as suggested by the supplier.

### ApoTox-Glo Triplex Assay

The assays were performed using a kit from Promega (Madison, WI). In brief, HDFs were seeded in 96-well plates. After an overnight incubation of cells, the medium was changed to the indicated glucose concentration. After 24 h of incubation, the viability/cytotoxicity reagent containing both GF-AFC substrate and bis-AAF-R110 substrate were added to the cells as suggested by the supplier. After about 1 hour incubation at 37 C, fluorescence was recorded at 400 nm excitation/505 nm emission for viability and 485 nm excitation/520 nm emission for cytotoxicity using a microplate reader for fluorescence (SPECTRA max 340PC, Molecular Devices, Sunnyvale, CA). Caspase-Glo 3/7 Reagent was further added to the cells and after ∼30 min of incubation at room temperature, luminescence was recorded using SpectraMax M2e (Molecular Devices). Numbers of viable, cytotoxic, and apoptotic cells were measured in triplicates.

### Transient Transfection and Dual-Luciferase Assays

All transient transfection assays were performed using FuGene6 transfection reagent (Roche, Indianapolis, IN) according to the manufacturer's instructions. In brief, sub-confluent cells were co-transfected with desired reporter plasmid (IFI16-luc; 1.8 µg DNA) along with pRL-TK plasmid (0.2 µg) as an internal control. 40–44 h after transfections of cells, firefly luciferase and *Renilla* luciferase activities were assayed using dual-luciferase reporter assay kit (Promega, Madison, WI). Relative luciferase activity was expressed as the ratio of the firefly luciferase and *Renilla* luciferase activities. Results are mean values of triplicate experiments and error bars represent standard deviation. The experiments were repeated at least two times.

## Results

### Glucose Restriction Activates the ATM/AMPK/p53 Pathway and Induces the Expression of IFI16 Protein

**Figure 1:**
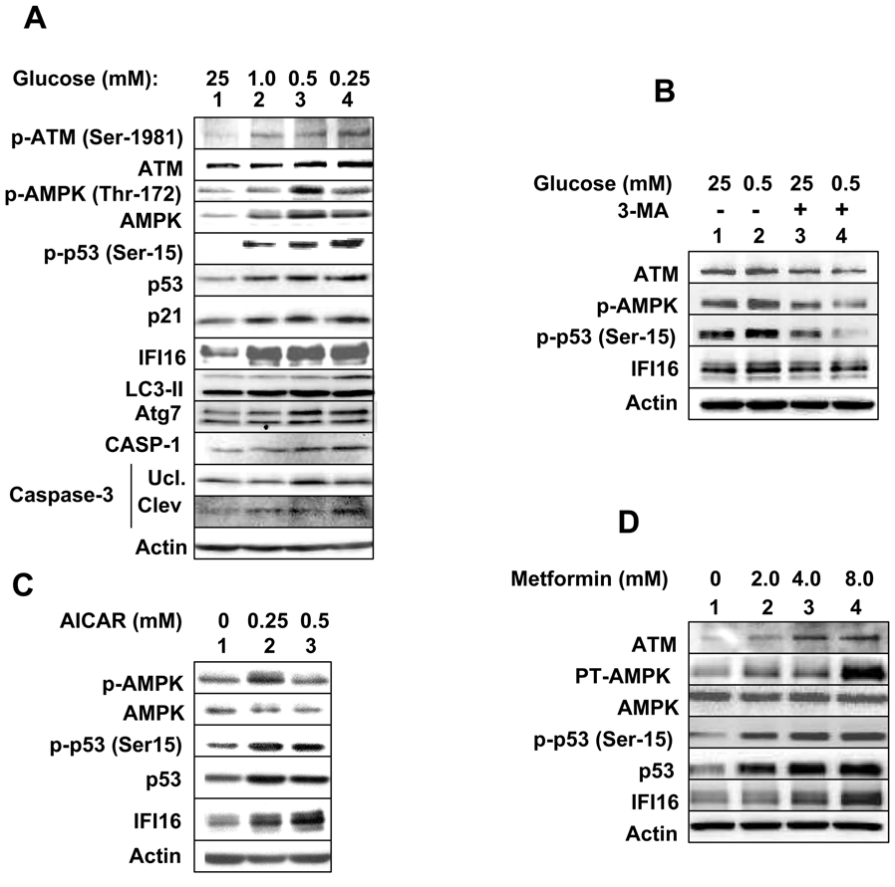
Incubation of HDFs under glucose restriction activates the ATM/AMPK/p53 pathway and induces the IFI16 expression. (**A**) Sub-confluent cultures of young WI-38 HDFs were incubated with the indicated concentrations (mM) of glucose in the medium supplemented with 10% fetal bovine serum for 18 h. At the end of the incubations, cells were lysed and equal amounts of proteins were analyzed by immunoblotting using antibodies specific to the indicated proteins. (**B**) Sub-confluent cultures of young WI-38 HDFs were incubated with the indicated concentrations (mM) of glucose in the medium without any treatment (lanes 1 and 2), with DMSO (lane 3) or with 3-methyladenine (5 mM; lane 4) for 18 h. At the end of the incubations, cells were lysed and equal amounts of proteins were analyzed by immunoblotting using antibodies specific to the indicated proteins. (**C**) Sub-confluent cultures of young WI-38 HDFs were incubated with the indicated concentrations (mM) of AICAR in the medium supplemented with 10% fetal bovine serum for 18 h. At the end of the incubations, cells were lysed and equal amounts of proteins were analyzed by immunoblotting using antibodies specific to the indicated proteins. (**D**) Sub-confluent cultures of young WI-38 HDFs were incubated with the indicated concentrations (mM) of metformin in the medium for 24 h. At the end of the incubations, cells were lysed and equal amounts of proteins were analyzed by immunoblotting using antibodies specific to the indicated proteins.

The ratio between AMP/ATP increases in HDFs that approach cellular senescence [35]. Moreover, treatment of mouse embryonic fibroblasts with low glucose increases the AMP/ATP ratio, activates AMPK, and results in phosphorylation of p53 at Ser-15 residue [11]. The phosphorylation activates and stabilizes p53 protein, thus, resulting in increases in the p53 protein levels [11]. Given that the expression of IFI16 protein is induced in old HDFs approaching cellular senescence [30] and the transcription of the *IFI16* gene is activated by wild type p53 [17], we incubated young HDFs with reduced glucose concentrations (1.0, 0.5, or 0.25 mM; the normal average glucose concentration in the human serum is ∼5 mM) to increase the AMP/ATP ratio to explore whether the expression of IFI16 is up-regulated by p53 that is activated by glucose restriction. As shown in Fig. 1A, the incubation of HDFs with 1.0 mM concentration of glucose resulted in the activating phosphorylation of ATM (Ser-1981), AMPK (Thr-172), and p53 (Ser-15) and increases in their protein levels. Furthermore, levels of the p53 target proteins, such as p21 and IFI16 (all three isoforms), also increased (∼2–3-fold) under glucose restriction conditions. Notably, the incubation of HDFs with glucose concentrations lower than 1.0 mM did not result in further appreciable increases in the levels of IFI16 protein, indicating that 1.0 mM concentration of glucose was sufficient to induce the expression of the IFI16 protein. Given that energetic stress that is induced by glucose restriction is known to induce autophagy and/or cell death [20]–[22], we also analyzed extracts for the expression levels of autophagy proteins. As shown in Fig. 1A, levels of the LC3-II and ATG7 proteins, markers of autophagy [36], increased with glucose restriction. Moreover, consistent with reduced cell viability under reduced glucose conditions, we also noted increases in levels of cleaved caspase-3 and caspase-1 (Fig. 1A), markers of cell death by apoptosis [37].

Our observations that WI-38 HDFs can initiate autophagy activity under glucose restriction prompted us to test whether treatment of cells with 3-methyladenine (3-MA), an inhibitor of autophagy [38], could inhibit the activation of the AMPK/p53 pathway and the induction of IFI16 expression under glucose restriction. As shown in Fig. 1B, the treatment of WI-38 HDFs with 3-MA reduced steady-state levels of the ATM kinase and the activating phosphorylation of both AMPK and p53 proteins (compare lane 4 with 2) under glucose restriction. Importantly, levels of the IFI16 protein did not increase appreciably after the 3-MA treatment under glucose restriction (compare lane 4 with 2). Notably, the difference in the IFI16 protein levels between the control (lane 2) and 3-MA-treated cells (lane 4) was relatively less because the treatment of cells with DMSO (the vehicle) alone increased the levels of IFI16 protein (data not shown). These observations revealed that treatment of HDFs with 3-MA inhibits glucose restriction-induced activation of the AMPK/p53 pathway and the induction of the IFI16 protein.

Treatment of cells with AICAR, an activator of AMPK, activates the AMPK/p53 pathway [11]. Therefore, we tested whether treatment of young WI-38 with AICAR could induce the expression of IFI16 protein. As shown in Fig. 1C, treatment of WI-38 HDFs with AICAR (0.25 or 0.5 mM), which increased the activating phosphorylation of AMPKα (at Thr-172) and p53 (at Ser-15), also increased the steady-state levels of IFI16 protein.

Treatment of cells with metformin, a drug used to treat patients with type 2 diabetes, also increases the AMP/ATP ratio [37]. Therefore, our above observations that glucose restriction of cells (or treatment with AICAR) induces the expression of IFI16 protein prompted us to investigate whether metformin could regulate the IFI16 expression in HDFs. As shown in Fig. 1D, treatment of HDFs with the indicated concentrations of metformin for 24 h increased levels of the IFI16 protein. Interestingly, the increase was associated with increases in the ATM protein levels and activation of AMPK and p53. These observations revealed that metformin-induced activation of the AMPK/p53 pathway can also induce the expression of the IFI16 protein. Together, these observations indicated that the energetic stress that is induced in WI-38 HDFs by glucose restriction, treatment of cells with AICAR or metformin can activate the ATM/AMPKα/p53 pathway and the activation is associated with increases in the steady-state levels of IFI16 protein and the induction of autophagy.

### Glucose Restriction Increases Levels of the IFI16 mRNA

**Figure 2:**
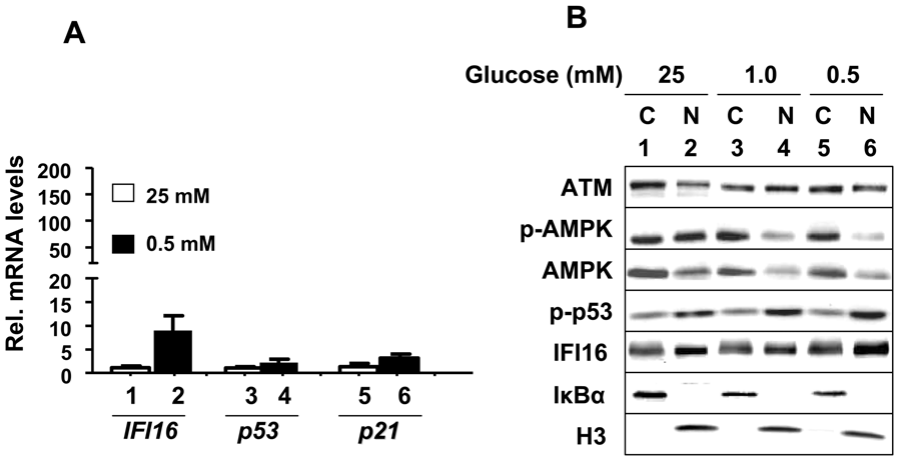
Glucose restriction increases steady-state levels of IFI16, p53, and p21 mRNA and increased levels of IFI16 protein are detected in the cytoplasmic and nuclear fractions. (**A**) Total RNA isolated from WI-38 cells incubated with the indicated concentration of glucose for 18 h was subjected cDNA synthesis, followed by quantitative real-time PCR using the TaqMan assay for the *IFI16*, *p53*, and *p21* genes. (**B**) Sub-confluent cultures of HDFs were incubated with the indicated concentrations of glucose in the medium for 18 h. At the end of the incubation, cells were fractionated into the cytoplasmic and nuclear fractions as described in methods. The fractions containing equal amounts of the protein were analyzed by immunoblotting using antibodies to the indicated proteins.

The *IFI16* gene is a transcriptional target of p53 [17]. Therefore, activation of p53 and increases in the IFI16 protein levels in the above experiments prompted us to test whether the levels of the IFI16 mRNA also increase in HDFs in response to energetic stress. As shown in Fig. 2A, steady-state levels of *IFI16* mRNA increased ∼10-fold in response to reduced glucose (0.5 mM) in the medium. Consistent with a previous report [14], levels of p53 and p21 mRNA also increased moderately.

### Glucose Restriction-induced IFI16 Protein is detected in the Cytoplasm and Nucleus

Given that sub-cellular localization of IFI16 protein depends on the cell type [27], we also assessed the sub-cellular localization of IFI16 protein in response to the energetic stress that is induced by glucose restriction. As shown in Fig. 2B, IFI16 protein was detectable in the cytoplasmic and nuclear fraction in control cells. Moreover, under glucose restriction, the IFI16 protein accumulated in the nucleus. Consistent with the previous reports [4], [5], we also detected the ATM protein in the cytoplasm and nucleus. Similarly, both AMPK and p53 were detected both in the cytoplasm and nucleus. However, glucose restriction resulted in the accumulation of AMPK primarily in the cytoplasm whereas the p53 protein in the nucleus. Given that the IFI16 and p53 proteins bind to each other [32], [33], the nuclear colocalization of the IFI16 and p53 proteins under glucose restriction conditions is consistent with their physical interactions.

### Glucose Restriction Decreases Cell Survival

**Figure 3:**
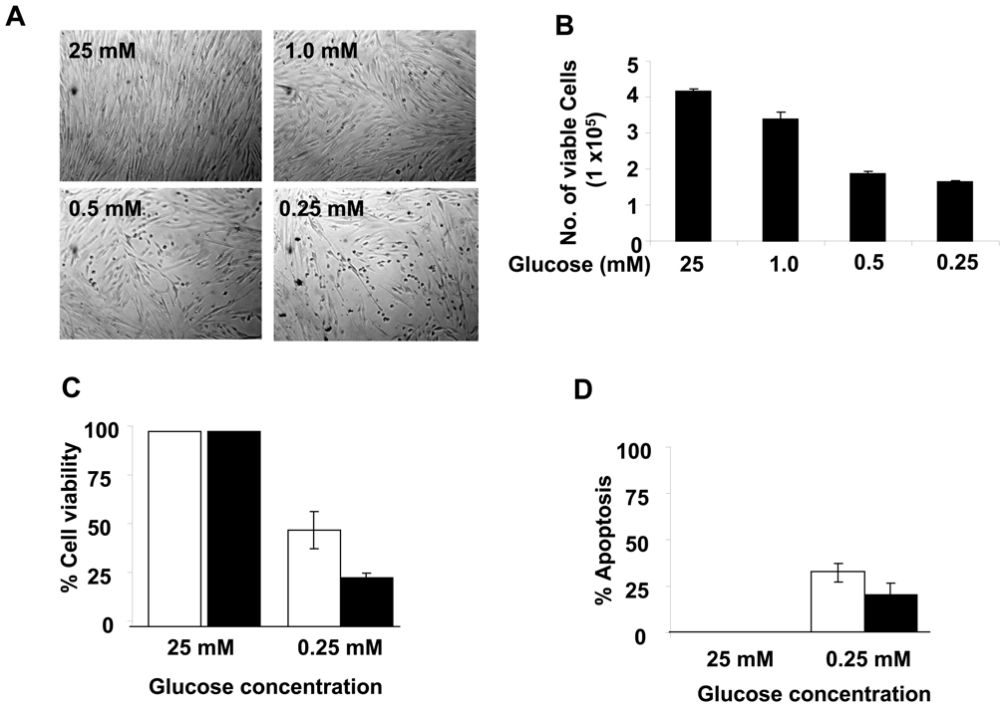
Glucose restriction decrease cell survival. (**A**) Sub-confluent cultures of young WI-38 HDFs were incubated with the indicated concentrations (mM) of glucose in the medium supplemented with 10% fetal bovine serum for 24 h. At the end of the incubations, cells were photographed using a phase contrast microscope. (**B**) At the end of the incubation of HDFs shown in the panel (A), the number of viable cells were counted (in triplicates) by Trypan Blue exclusion method using the Countess Automated Cell Counter (Invitrogen). (**C** and **D**) HDFs (in triplicates) were grown in 96-well plates and cells were incubated at the indicated concentrations of glucose for either 24 h (white bars) or 48 h (black bars). At the end of the incubations, cell viability and the extent of apoptosis were determined using ApoTox-Glo Triplex Assay (Promega) as described in the method. Experiments were repeated three times, which gave similar results.

Glucose restriction in cells is known to induce autophagy [20], [21], [39] and cell death by apoptosis [20]–[22]. Therefore, increased levels of the LC3-II and ATG7 proteins, biomarker for autophagy [36], and increased cleavage of caspase-3 and caspase-1, markers for cell death by apoptosis (Fig. 1A), prompted us to investigate whether the energetic stress in HDFs reduces cell viability. As shown in Fig. 3A, incubation of cells with reduced glucose concentrations resulted in morphological changes consistent with reduced cell viability (Fig. 3B and C). Furthermore, we noted that the incubation also increased cell death by apoptosis (Fig. 3D). Together, these observations revealed that glucose restriction in WI-38 HDFs under our experimental conditions induced autophagy and cell death.

### Glucose Restriction-induced Increase in the IFI16 Protein Levels is ATM-Dependent

**Figure 4:**
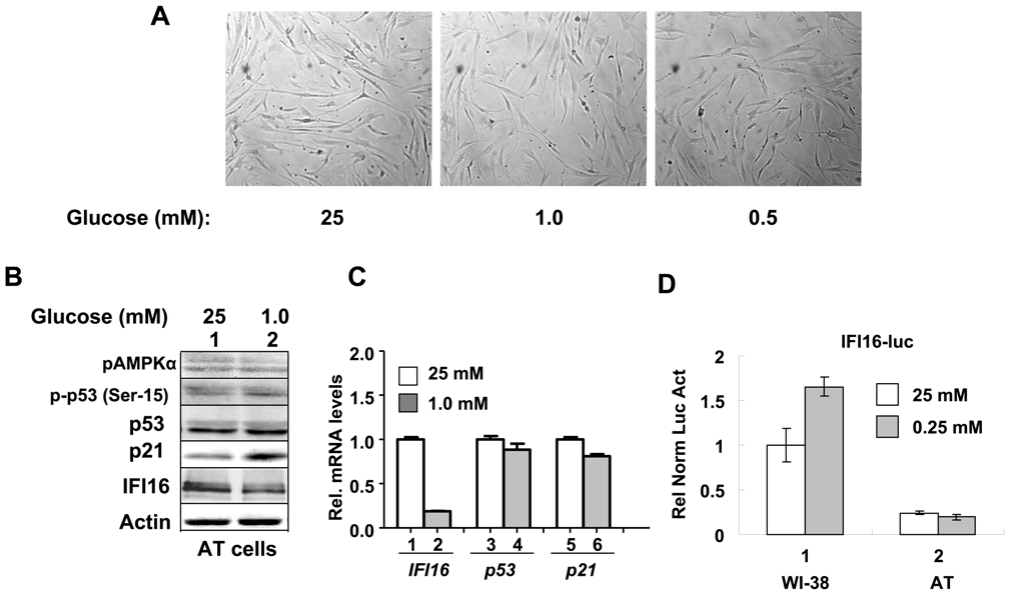
Glucose restriction-induced activation of the AMPK/p53 pathway and increase in the IFI16 protein levels are ATM-dependent. (**A**) Sub-confluent cultures of young AT HDFs were incubated with the indicated concentrations (mM) of glucose in the medium supplemented with 10% fetal bovine serum for 18 h. At the end of the incubations, cells were photographed using a phase contrast microscope at a lower magnification. (**B**) Sub-confluent cultures of young AT HDFs were incubated with the indicated concentrations (mM) of glucose in the medium supplemented with 10% fetal bovine serum for 18 h. At the end of the incubations, cells were lysed and equal amounts of proteins were analyzed by immunoblotting using antibodies specific to the indicated proteins. (**C**) Total RNA isolated from AT HDFs incubated with the indicated concentration of glucose for 18 h was subjected cDNA synthesis, followed by quantitative real-time PCR using the TaqMan assay for the *IFI16*, *p53*, and *p21* genes. (**D**) Sub-confluent cultures of young WI-38 or AT HDFs were transfected with the IFI16-luc reporter plasmid (1.8 µg) along with a second reporter pRL-TK (0.2 µg) plasmid as an internal control. After transfections, cells were incubated with the indicated concentrations of glucose. 40–44 h after transfections, firefly luciferase and *Renilla* luciferase activities were assayed using dual-luciferase reporter assay kit. Relative luciferase activity was expressed as the ratio of the firefly luciferase and *Renilla* luciferase activity. The numbers indicate fold change in the activity of the firefly luciferase. The experiments were repeated two times with essentially similar results.

Activation of ATM protein kinase by glucose restriction in HDFs has not been reported previously. Therefore, our observations that glucose restriction (Fig. 1A) increases the activating phosphorylation of ATM protein kinase prompted us to test whether the activation of energetic stress in HDFs is the ATM-dependent. As shown in Fig. 4A, incubation of young (passage 6) AT HDFs under reduced glucose concentrations (1.0 or 0.5 mM) for 24 h did not result in any appreciable morphological changes. Moreover, we noted accumulation of cells primarily in the G_1_ phase of the cell cycle at the expense of the S-phase (data not shown). Significantly, we could not detect any appreciable increase in the activating phosphorylation of AMPK or p53 under reduced glucose concentrations (1.0 mM; Fig. 4B). Notably, the levels of IFI16 protein did not increase appreciably and levels of IFI16 mRNA decreased >70% under reduced glucose conditions (Fig. 4C). Accordingly, no measurable changes in the mRNA levels of p53 and p21 were evident. Moreover, the activity of the IFI16-luc reporter, the transcription of which is driven by the 5′-regulatory region of the *IFI16* gene [17], was not stimulated by the reduced glucose concentration (0.25 mM) in the medium (Fig. 4D). Together, these observations suggested that AT HDFs are defective in glucose restriction-induced activation of the AMPK/p53 pathway and the induction of the *IFI16* expression.

### Glucose Restriction-induced Expression of IFI16 Protein Contributes to Activation of the ATM/AMPK/p53-pathway

**Figure 5:**
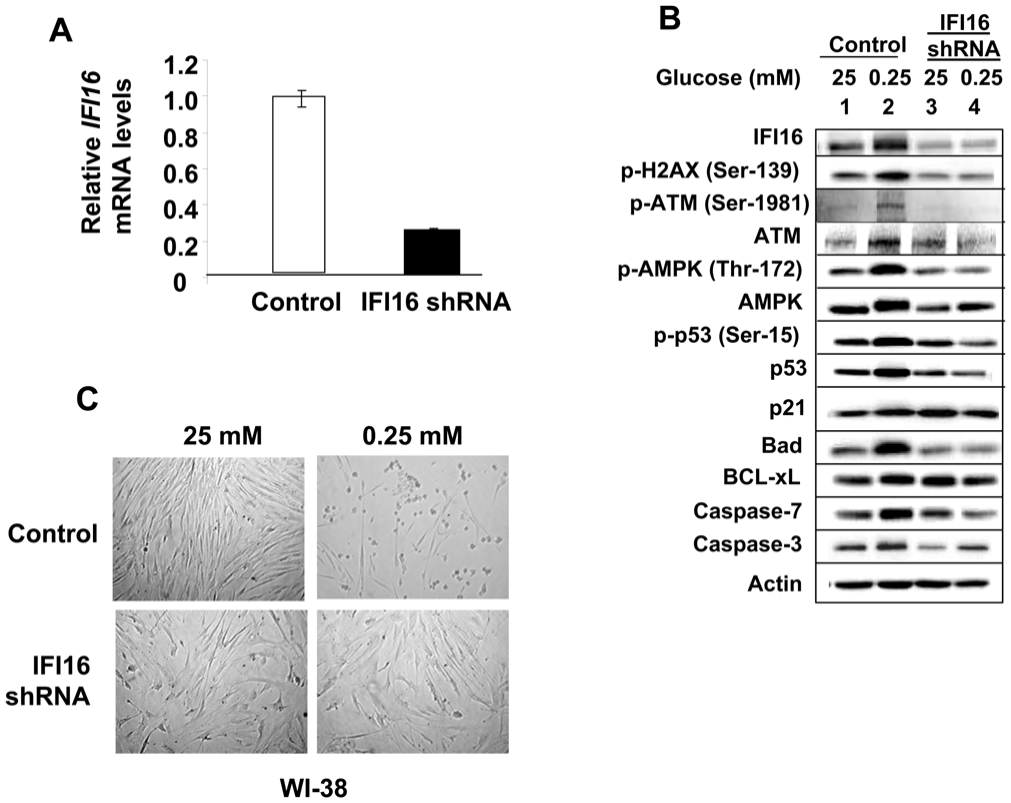
The expression of IFI16 protein is required for the activation of the ATM/AMPK/p53 pathway in response to glucose restriction. (**A**) Total RNA isolated from WI-38 HDFs either infected with control or IFI16 shRNA lentivirus particles was subjected cDNA synthesis, followed by quantitative real-time PCR using the TaqMan assay for the *IFI16* gene. (**B**) Sub-confluent cultures of young WI-38 HDFs either infected with control (Vector) or IFI16 shRNA lentivirus particles (shIFI16) were incubated with the indicated concentrations (mM) of glucose in the medium supplemented with 10% fetal bovine serum for 18 h. At the end of the incubations, cells were lysed and equal amounts of proteins were analyzed by immunoblotting using antibodies specific to the indicated proteins. (**C**) Sub-confluent cultures of young WI-38 HDFs either infected with control (top panel) or IFI16 shRNA lentivirus particles (lower panel) were incubated with the indicated concentrations (mM) of glucose in the medium supplemented with 10% fetal bovine serum for 18 h. At the end of the incubations, cells were photographed using a phase contrast microscope.

To examine the potential role of the IFI16 protein in the glucose restriction-induced activation of the ATM/AMPK/p53 pathway, we knockdown the expression of IFI16 protein in the young WI-38 HDFs (Fig. 5A and B) and compared the activation of the pathway by glucose restriction between the control cells and after the knockdown of *IFI16* expression. As shown in Fig. 5B, the knockdown (∼70% reduction) of IFI16 expression in WI-38 HDFs, under reduced glucose conditions, decreased the activating phosphorylation of ATM, AMPK, and p53 (Fig. 5B). Notably, the basal levels of caspase-3 also decreased in HDFs with reduced levels of the IFI16 protein and the energetic stress resulted in only moderate increases in the levels. In addition, basal levels of pro-apoptotic protein Bad, a transcriptional target of p53, decreased whereas basal levels of anti-apoptotic protein BCL-xL increased in cells with the reduced IFI16 protein levels. Furthermore, no increase in levels of Caspase-7 was evident under energetic stress in cells with reduced IFI16 protein levels. Consistent with these observations, the knockdown of IFI16 expression in HDFs also decreased the morphological changes (Fig. 5C) that were associated with reduced cell survival under reduced glucose concentrations (Fig. 3). Together, these observations revealed that the IFI16 protein contributes to the activation of the ATM/AMPK/p53 pathway during the glucose restriction and the reduced levels of the IFI16 protein in cells increase cell survival by modulating the expression of the p53-regulated apoptosis-regulatory proteins.

### IFI16 Protein Potentiates the p53-mediated Transcriptional Activation

**Figure 6:**
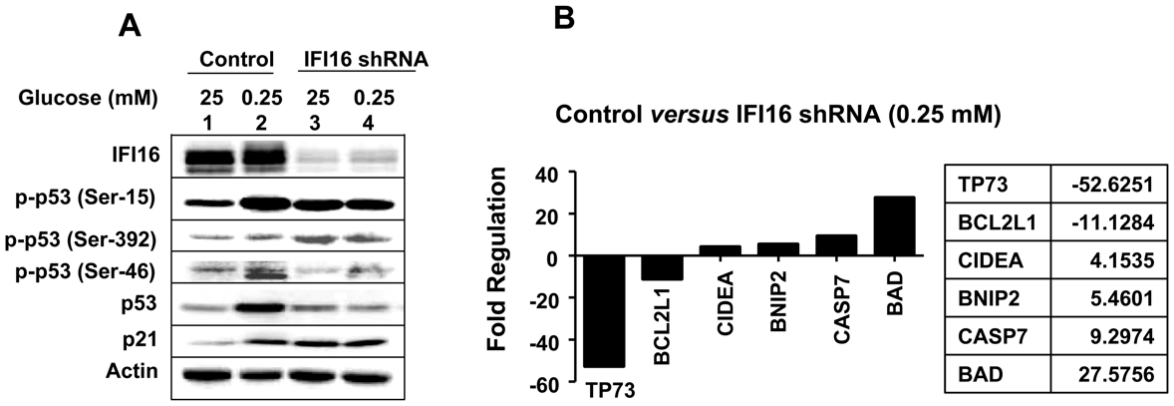
IFI16 protein potentiates the p53-mediated transcriptional activation through the regulation of phosphorylation. (**A**) Sub-confluent cultures of young WI-38 HDFs either infected with control (Vector) or IFI16 shRNA lentivirus particles (shIFI16) were incubated with the indicated concentrations (mM) of glucose in the medium supplemented with 10% fetal bovine serum for 18 h. At the end of the incubations, cells were lysed and equal amounts of proteins were analyzed by immunoblotting using antibodies specific to the indicated proteins. (**B**) Young WI-38 HDFs either infected with control or IFI16 shRNA lentivirus particles were incubated with medium containing reduced glucose concentration (0.25 mM) for 18 h. At the end of the incubation, total RNA was isolated and subjected cDNA synthesis. The cDNA preparation was used for RT^2^ Profiler PCR Array for human apoptosis genes as described in Methods. Genes whose expression was increased or decreased more than 4-fold are indicated.

Phosphorylation of p53 at Ser-15 or Ser-46 is associated with energetic stress-induced metabolic checkpoint and induction of apoptosis [40], [41]. Moreover, the IFI16 protein alters the phosphorylation of p53 in human MCF-7 [31] and endothelial [42] cells. Therefore, we investigated whether the knockdown of IFI16 expression in HDFs affects phosphorylation of p53 protein under glucose restriction. As shown in Fig. 6A, glucose restriction increased IFI16 protein levels (compare lane 2 with 1). Notably, the increase in the IFI16 protein levels in lane 2 is moderate due to reduced protein levels (compare actin protein levels between lane 2 and 1). Importantly, the knockdown of IFI16 expression in young WI-38 HDFs resulted in increased basal phosphorylation of the p53 at the Ser-392 and reduced basal phosphorylation of p53 at Ser-46. Moreover, the energetic stress, which increased p53 phosphorylation at Ser-46 in control cells, was inhibited in HDFs with reduced IFI16 protein levels. Interestingly, phosphorylation of p53 at Ser-15 did not increase under reduced glucose conditions in HDFs with reduced IFI16 protein levels. Notably, the p53 protein levels did not increase in HDFs with reduced IFI16 protein levels under glucose restriction. Accordingly, we noted changes in the steady-state levels of mRNAs encoded by the p53 target genes between control HDFs and HDFs with the reduced IFI16 protein levels under the energetic stress (Fig. 6B). We found that the steady-state levels of mRNAs corresponding to the *TP73* and *BCL2L1* genes were reduced >10-fold. In contrast, the steady-state levels of *CIDEA*, *BNIP2*, *CASP7*, and *BAD* mRNAs were increased 4–27-fold. These observations suggested that the increased levels of the IFI16 protein under glucose restriction modulate the p53-mediated cell survival by modulating the phosphorylation of p53 and the expression of its target genes.

## Discussion

**Figure 7:**
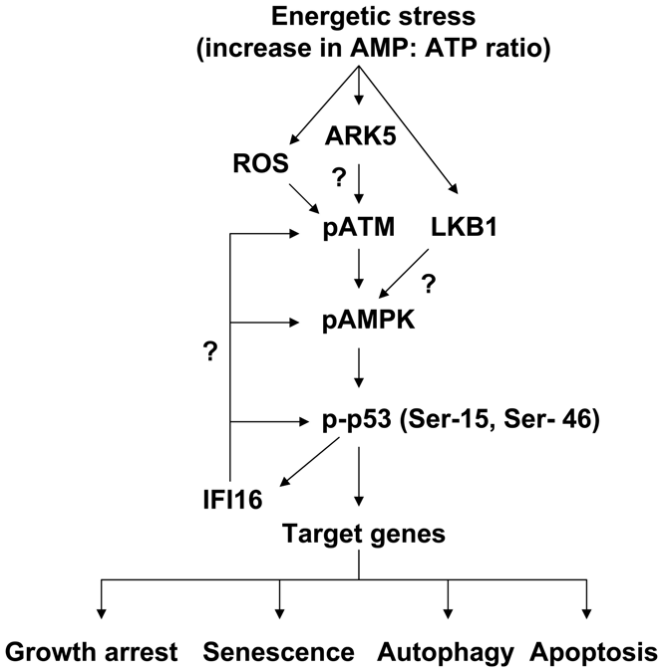
Proposed model for the regulation and role of the IFI16 protein in glucose restriction-induced activation of the ATM/AMPK/p53 pathway and autophagy.

In the current study, we investigated the regulation and role of the IFI16 protein, a transcriptional target of the p53 and a growth suppressor [17], [27], in energetic stress-induced signaling in HDFs. We found that the energetic stress due to glucose restriction, treatment of HDFs with AICAR or metformin activated the ATM/AMPK/p53 pathway. Interestingly, the activation of the pathway was associated with increases in steady-state levels of IFI16 protein (Fig. 1) and mRNA (Fig. 2A). The increased levels of IFI16 protein were detected in the cytoplasmic as well as in the nuclear fraction (Fig. 2B). Moreover, the increased levels of the IFI16 protein were associated with autophagy (Fig. 1A) and decreased cell viability (Figs. 1 and 3). In contrast, the knockdown of IFI16 expression in HDFs under glucose restriction inhibited the activation of the pathway and also increased cell survival (Fig. 5). These observations are novel and are consistent with the idea that the disruption of energetic stress-induced checkpoint through the loss of the ATM function, as is the case in AT cells, or the loss of p53 or IFI16 function, as is the case in certain human cancers, may provide a growth advantage to cells under energetic stress. Moreover, these observations also support the idea that the energetic stress-induced increased levels of the IFI16 protein in cells contribute to autophagy (Fig. 7).

Depending upon glucose restriction-induced energetic stress levels, autophagy in cells is thought to promote either cell survival or induce cell death [20]–[23]. Interestingly, we noted that glucose restriction consistently reduced survival of WI-38 HDFs in several independent experiments. We also noted cleavage of both caspase-3 and caspase-1 (Fig. 1A) with reduced cell survival (Fig. 3), suggesting some cell death by apoptosis in glucose restricted WI-38 HDFs. Given that studies indicate p53-dependent apoptosis [19] as well as atypical form of apoptosis [22] in glucose restricted cells, it is likely that reduced cell survival under our experimental conditions is due to both apoptosis (in an early stage when energy is still available to cells) and necrosis. Further work is in progress to understand the molecular mechanisms by which glucose restriction induces autophagy and cell death in HDFs.

The onset of cellular senescence is thought to protect against the initiation of tumor formation in response to certain cellular stresses, including the genotoxic and energetic stress [43]. It is known that environmental factors that place oxidative stress on cells promote the early onset of cellular senescence and increase the AMP: ATP ratio significantly (∼30-fold) [35], which results in energetic stress and activation of a stress pathway involving the AMPK and p53 [11]. Our observations that energetic stress that is induced by glucose restriction (or treatment of cells with AICAR or metformin) can activate the ATM/AMPK/p53 pathway in HDFs and the activation is associated with increases in the IFI16 protein levels make it likely that the increased levels of IFI16 protein in cells contribute to cellular senescence associated cell growth arrest.

Others have reported that AMPK activate a nutrient-sensitive signaling pathway that initiates p53-dependent cell-cycle arrest during times of energy deficiency [11]. Thus, p53 serves as a metabolite sensor and, depending upon cell type and the strength of the stress, coordinates cell survival, resulting in reversible cell-cycle arrest, cellular senescence, or reduced cell viability [11], [14].

In response to certain cellular stresses, p53 is phosphorylated on several amino acid residues [40], [41]. These residues include Ser-15, Ser-20, and Ser-46. Phosphorylation of p53 protein at Ser-46 after severe DNA damage results in apoptotic cell death. Therefore, it has been proposed that the stress-induced phosphorylation of p53 at Ser-46 is one of the critical events for commitment of cell fate into apoptotic cell death [40], [41]. Given that IFI16 protein binds to p53 and potentiates the p53-mediated transcription [32], [33], our observations that the knockdown of IFI16 expression in HDFs reduced phosphorylation of p53 at Ser-46 in response to the energetic stress (Fig. 6) make it likely that the IFI16 protein regulates cell survival of HDFs under glucose restriction by regulating the phosphorylation of p53 and, thus, altering the expression of its target genes that encode for apoptosis regulating proteins (Fig. 6).

Activation of the ARK5, a relatively new member of the human AMP-activated protein kinase (AMPK) family, during nutrient starvation is shown to support cell survival [44]. In addition, the activated ARK5 protein kinase can phosphorylate the ATM and p53 proteins [44]. Therefore, our observations that under glucose restriction the ATM is phosphorylated at Ser-1981 in HDFs are consistent with the possibility that energetic-stress induced kinases, such as ARK5, may activate both ATM and p53. Further work will be needed to test this possibility.

In response to oxidative damage, cellular DNA undergoes oxidative damage, which is considered as one of the key factor in cellular aging [45]. Cellular DNA damage (particularly the induction of DSBs) results in activating phosphorylation of ATM at Ser-1981 and histone H2AX at Ser-139 [46]. The phosphorylation of these two proteins serves as marker for oxidative DNA damage. Cells under normal culture conditions (20% O_2_) exhibit basal oxidative damage as indicated by the constitutive phosphorylation of both ATM and H2AX [46]. Moreover, the extent of phosphorylation of these proteins reflects the extent of oxidative DNA damage. Therefore, our observations that the knockdown of IFI16 expression in young HDFs reduced the constitutive phosphorylation of both H2AX and ATM (Fig. 5B) are consistent with our previous observations that the increased levels of IFI16 protein in old HDFs are associated with the activation of p53, increased expression of p21, and the onset of cellular senescence [30]. Furthermore, we noted that incubation of young WI-38 HDFs at lower (1%) O_2_ levels reduced steady-sate levels of the IFI16 mRNA and protein by 50–70% within 24 h of incubation (data not shown). Therefore, it is likely that incubation of WI-38 HDFs under our culture conditions (20% O_2_) and glucose restriction-induced stress both contribute to the increased levels of the IFI16 protein.

Cells respond to oxidative stress and energy depletion with the induction of autophagy [19], [39]. Therefore, our observation that increases in the IFI16 protein levels in HDFs in response to glucose restriction are associated with increases in levels of the LC3-II and ATG7 proteins, markers of the induction of autophagy [36], raise the possibility that the increased levels of IFI16 protein in HDFs contribute to glucose deprivation-induced increases in reactive oxygen species (ROS) and, thereby, depletion of ATP and cell death. Accordingly, we noted that the knockdown of IFI16 levels in HDFs reduced glucose deprivation-induced increase in the phosphorylation of H2AX and ATM, and cell death (Fig. 5). Moreover, these results are also consistent with the idea that the loss of IFI16 expression, as found in human cancers [27], could provide a survival advantage to tumor cells in microenvironments with glucose restriction.

In summary, our observations demonstrate a role for the IFI16 protein in the energetic stress-induced activation of the ATM/AMPK/p53 pathway (Fig. 7) and autophagy. At present, it is not clear how ATM protein kinase is phosphorylated in response to the energetic stress. Given that reduced levels of glucose are known to generate reactive oxygen species (ROS) [47], it is likely that the oxidative stress activates the ATM kinase. Notably, glucose deprivation of cells and their ability to survive under low glucose conditions can drive the acquisition of *KRAS* pathway mutations in human tumors [48]. In this context, it is interesting to note that the loss of *IFI16* expression in human colon cancer cell lines is associated with *KRAS* mutations [21]. Therefore, further studies will be needed to examine the role of IFI16 protein in the energetic-stress-induced metabolic pathways, autophagy, cell survival, and the development of cancers.
